# 

**Published:** 1850-12

**Authors:** 


SELECTED MATTER.—We have bhen compelled to omit a great deal of selected matter and several editorials prepared for this number.

### INDEX TO VOL. VI.

[THIRD SERIES.]

A

Acid, nitric, in asthma............439
Acquired idiosyncrasy ..............16
Adjuster, Jarvis's, in the reduction of dislocations, and treatment of fractures ..........................331
Ague treated by a single dose of quinine ..........................108
American Medical Association......85
A memoir on mercury in typhoid fever..............................509
Anatomical drawings ..............365
Anderson's case of twins..........19
Aneurism treated successfully by compression ......................79
A new medical school .............89
Apology to the readers of the Medical Journal.................184
A report on typhoid fever, by W. L. Sutton, M.D.......185, 277, 369
Ascites, clinical convictions respecting.........................110
A singular case of pregnancy........7
Asthma treated with nitric acid....439

B

Bartlett's Prof., testimony to the value of Peruvian bark .........477
Becquerel's memoir on mercurial treatment of typhoid fever......509
Bell's, T. S., notes on miscellaneous subjects contained in Foreign and home medical journals...8
Bell's, T. S., brief notes on cholera in Louisville, 1850............99
Bell, T. S., on watering streets ...327
 on Jarvis's adjuster....331
 on quinine and its various preparations ....461
Books received....................276
Brequet on quinine................501

C

Calculus, formed on a curved hair, extracted through the umbilicus.443
Case of monstrosity..................1
 of gun-shot wound...........131
 of pregnancy....................7
 of premature labor artificially induced at the 7th month ..121
 of excission of the uterus, by Prof. Paul F. Eve ........401
Case in which the urachus remained open, and a ring-shaped calculus was extracted thro' the umbilicus...............443
 of uterine pregnancy of over twenty years' continuance .449
Cases of asthma successfully treated with nitric acid..............439
Catarrhal pneumonia ...............108
Changes in medical schools ..178, 275
Charcoal in nervous gastro-intestinal affections ....................15
Chemical instruction...............182
Child, spontaneous expulsion of,..145
Children, incontinence of urine in.266
 pneumonia in..........76
Chloroform during parturition ....130
Chloroform, febrifuge and anti-periodic properties of...........265
Chloroform.........................366
Chloroform in puerperal convulsions..............................441
Chlorini liquor, external employment of............................452
Cholera statistics ...................15
 quinine used in, by Drs. C. W. Bell and C. L. Lyle..............490, 493
 brief notes on its appearance in Louisville, Ky., 1850 ...................99
Chronic diarrhea...................460
Cities, mortality of ...............266
Clinical convictions respecting ascites ............................110
Clinical surgery of Prof. Eve.....457
Close of Volume Sixth.............546
Cod-liver oil, probable danger from its use.......................268
Cod-liver oil.........................73
Cod-liver oil in tertiary symptoms..16
Compression in the treatment of aneurism..........................448
Condition of the ovaries soon after menstruation .................139
Congestive chill, Senac's description of..........................487
Congestive fever, quinine in.......486
Convulsions, puerperal, chloroform in..........................441

Correspondence, foreign, of the Western Journal of Med. ...93, 184
Cow-pock, spontaneous reproduction of ....84
Croup treated by topical applications of nitrate of silver ....18
D
Danger from the use of cod-liver oil....268
Death of Dr. Troost....269
Diarrhea, chronic ....460
Diseased aortic valves ....73
Dislocation of the lower jaw....105
Drawings, anatomical ....365
Dysmenorrhea....148
E
Effects, therapeutical of turpentine ....132
Elston's, Dr. use of exhausted tumbler ....494
Emission of urine from ectopia of the bladder ....83
Emmenagogue properties of polygala senega....445
Epidemics, why they rage at night ....265
Epilepsy, treatment of ....17
Essay on physiology or chemistry, premium for....86
Essay, prize, on nostrums ....87
Eve, Prof. Paul F., appointment of in Louisville Med. School ...361
Eve's clinical surgery ....457
Eve's, Prof., card to the committee on surgery of the Am. Med. Association, and to the surgeons of the United States....541
Excission of the uterus, by Prof. Paul F. Eve ....401
Expulsion, spontaneous, of the child....145
External employment of liquor chlorini ....452
Extraction of a bullet from the bladder....131
Exudation, tubercular, into the lungs ....73
F
Febrifuge and antiperiodic properties of chloroform....265
Febrifuge, new, discovered by A. Seguin....472
Fever, typhoid report on, by W. L. Sutton....185, 277, 369
Fever, typhoid M. Becquerel's memoir on the mercurial treatment of ....509
Fever, typhoid Dr. Sutton on ....273
Flint's, Austin Prof. appeal to the profession as chairman of the committee on practical medicine, of the American Medical Association ....528
Foreign correspondence..93, 184, 455
Fractures of the head, statistics of mortality from ....128
Fractures, treatment of with Jarvis's adjuster....331
Fungus hematodes ....275
G
Garret's case of monstrosity ....1
Genital organs, irritation of allayed by Lupulin ....451
Gleet and its treatment....83
Gun-shot wound of the bladder ...131
H
Head, mortality from fractures of..128
Heart, sounds musical ....73
Hematodes, fungus....275
Hemorrhoids internal, treatment of 257
Homœopathy, Latta on ....182
Hydrostatic test....113
Hygiene ....8
I
Idiosyncrasy acquired....16
Incontinence of urine in children..266
Inflammation, nitrate of silver in ...13
Insanity from the use of chloroform during parturition ....130
Instruments, surgical ....460
Internal hemorrhoids ....257
Intestinal affections, charcoal in....15
J
Jarvis's adjuster....272
Jarvis's adjuster in the reduction of dislocations and treatment of fractures ....331
Journal of Medicine, foreign correspondence of ....93
Journal of Medicine, apology to the readers of....184
Journal of Medicine, New Hampshire....272
L
Labor, premature artificially induced at the seventh month....121
Lapis divinus in otorrhea and diarrhea....460
Lancet, the Western and Memphis School advertisement ....366
Laryngotomy and tracheotomy....442
Latta, Dr. on homœopathy ....182
Lecture terms, prolonged....362
Libel, Leomis's trial for ....274
Liberty, the largest ....367
Liquid muriate of opium ....488
Liquor chlorini, external use of...452
Loomis, trial for libel ....274
Louisville, University of....361

Lungs, tubercular exudation into...73
Lupulin in irritability of the genital organs ....................451
Lyle's, Dr. cases of cholera ..491, 493
use of exhausted tumbler................493

M

Medical Association, American .....85
chemistry, premium for essay on..............86
school, a new one .........89
school, Ohio ..............181
schools, changes in..178, 275
schools....................183
schools, New York .......180
teaching in New York ...364
school, Transylvania......453
Association, American proceedings of.........457
Association, Southern Central, N. York.......273
Professors, puffing of....458
Medicine, practical ................365
practical, committee of appeal of Prof. Flint ..528
in Spain ...............149
Memphis school....................366
Memphis school. advertisement of.366
Menstruation, state of the ovaries soon after ....................139
Mercurials in typhoid fever ........509
Method of depriving quinine of its bitterness.......................82
Miscellaneous subjects contained in Foreign and Home Medical Journals, notes on.................8
Mortality from fractures of the head, statistics of...............128
Mortality of cities ..................266
Mucous pneumonia in children ...108
Muriate of opium, liquid ..........488
Musical heart sounds ...............73

N

New remedies ......................274
Nitric acid in asthma ..............439
Nostrums, prize for essay on ......87
Notes on miscellaneous subjects contained in Foreign & Home Journals ..........................8

O

Obituary ..........................181
Obstetricy, use and advantages of opium in .......................114
Ohio Medical school..............181
On gleet and its treatment.........83
On the emission of urine, as observed in ectopia of bladder ......83
On a case of spontaneous reproduction of cow-pock............84
On the dislocation of the lower jaw 105
On the treatment of ague by a single dose of quinine ............108
On mucous or catarrhal pneumonia in children ..................108
On phlebitis .......................113
On the hydrostatic test............113
On the use and advantages of opium in obstetrics................114
On the statistics of mortality from fractures of the head............128
On the alleged frequency of ulceration of the os and cervix uteri..258
On incontinence of urine in children .............................266
On watering streets................327
On the emmenagogue properties of Polygala senega .............445
On the history of quinine, the preparations of that article, and their use in medicine ...........461
Opium, liquid muriate of..........488
Opium in obstetrics................114
Osborne's singular case of pregnancy at the full period ...........7
Os uteri, alleged frequency of ulceration in.......................258
Otorrhea, lapas divinus in.........460
Ovaries, condition of soon after menstruation ....................139

P

Parturition, insanity from the use of chloroform in.................130
Pelletier and Caventou, their merits ....................474, 504
Phlebitis ...........................113
Phthisis Pulmonalis ...............73
Physiology, premium for essay on..86
Pneumonia in children.............76
Polygala senega as an emmenagogue..............................445
Popliteal space, aneurism of treated by compression..............79
Power of lupulin in allaying irritability of the genital organs ....451
Practical medicine.................365
Practice of obstetrics, opium in ...114
Pregnancy, singular case of ........1
Pregnancy, uterine of over twenty years' standing..................449
Premature labor artificially induced 121
Premium for an essay on physiology, &c. ..........................86
Prize essay on nostrums ...........87
President of the Philadelphia county medical society ..........271
Probable danger from use of cod-liver oil ..........................268
Proceedings of the American Medical Association ............151, 457

Prof. Paul F. Eve's clinical surgery ....457
Prolonged lecture terms ....362
Prussiate of iron in dysmenorrhea .148
Puffing of medical professors ....450

Q

Quackery, arts of ....458
Qunine, method of depriving it of its bitterness ....82
Quinine, single dose of in ague ...108
in the treatment of dysmenorrhea ....148
history of ....461
powers of discovered by the Jesuits ....462
importance of in atumnal fever ....466
Armand Seguin's substitute for ....472
sulphate of discovered by Pelletier & Caventou ..474
medical testimony of its value ....476
relations of to disease ....478
prompt use commended .479
during the exacerbation of fever ....481, 482
use of in one large dose in intermittent fever ...483
its continued use for sometime after the paroxysms are broken ....484
in remittent fever ....485
in congestive fever ....486
in cholera ....489
in cholera, successfully used by Dr. Lyle ....490
in rheumatism ....495
in neuralgia ....496
in intermittent affections of the eye, &c. ....497
in obstinate hiccup ....499
in itermittent asthma ....498
in engorgement of spleen 499
in yellow fever ....500
in large doses ....501
M. Brecquett's experiments and deductions respecting ....501
substitute for ....503
to be made by chemistry, fallacious hope ....504
invaluable on account of the adulterations of Peruvian bark, and the difficulty of detecting them ....505
adulterations of ....505, 507
incompatibilities of ....507
Prof. A. Flint's use of— note ....508

R

Register of Medicine and Pharmacy ....362
Registration ....546
Remarks on vermifuges ....141
Report of a case of premature labor ....121
Report on typhoid fever by W. L. Sutton, M. D. ....185, 277, 369
Report of the trial of H. N. Loomis, for libel ....274

REVIEWS.

Medical Jurisprudence. By Alfred S. Taylor, F. R. S., member of the Royal College of Surgeons; and Professor of Medical Jurisprudence and Chemistry in Guy's Hospital. With notes and additions by R. Eglesfeld Griffith, M. D., &c., .... 21
Southern Medical Reports; consisting of General and Special Reports on the Medical Topography, Meteorology, and Prevalent Diseases in the following States: Louisiana, Alabama, Mississippi, N. Carolina, S. Carolina, Georgia, Florida, Arkansas, Tennessee, Texas. Edited by E. D. Fenner, M. D., of New Orleans; member of the American Medical Association, &c., .... 26
A Treatise on the Etiology, Pathology, and Treatment of Congenital Dislocations of Head of the Femur. Illustrated with Plates. By John Murray Carnochan, M. D., Lecturer on Operative Surgery with Surgical and Pathological Anatomy .... 29
The American Medical Formulary, based upon the United States and British Pharmacopeias. Including also, numerous standard Formula, derived from American and European authorities. Together with the Medical Properties and Uses of Medicines; Poisons, their Antidotes, Tests, &c. Designed for the Medical and Pharmaceutical Student. By J. Reese, M. D., Lecturer on Materia Medica and Therapeutics in the Philadelphia Medical Institute, &c. .... 38
On the Use and Abuse of Alcoholic Liquors, in Health and Disease. Prize Essay. By William B. Carpenter, M. D., F. R. S., F. G. S., author of "Principles of Human Physiology," &c., &c. .... 39

Essays on the Puerperal Fever and other Diseases peculiar to Women. Selected from the Writings of British Authors previous to the close of the Eighteenth Century. Edited by Fleetwood Churchill, M. D., M. R. I. A., Hon. Fellow of the College of Physicians of Ireland; Corresponding Member of the American National Institute, &c. ..................... 41
Transactions of the Medical Society of the State of N. York. During its Annual Session, held at Albany, Feb. 5, 1850............. 47
A Systematic Treatise, Historical, Etiological, and Practical, on the Principal Diseases of the Interior Valley of North America, as they appear in the Caucasian, African, Indian, and Esquimaux varieties of its Population. By Daniel Drake, M. D. .........228, 350, 408
The Principles and Practice of Dental Surgery. By Chapin A. Harris, M.D., D.D.S., Professor of Principal and Practice of Dental Surgery in the Baltimore College; Fellow of the American Society of Dental Surgeons; Member of the American Med. Association, etc. etc. Fourth edition: revised, modified, and enlarged. With 200 illustrations ...........516
Observations on certain of the Diseases of young Children. By Charles D. Meigs, M.D., Professor of the Diseases of Women and Children, in the Jefferson Medical College, at Philadelphi, etc. etc. ......................519
Of the Causes, Nature, and Treatment of Palsy and Apoplexy: of the forms, seats, complications, and morbid relations of Paralytic and Apoplectic Diseases. By J. Copland, M.D., F.R.S., etc.....521
Human Physiology. By Robley Dunglison, M.D., Professor of the Institutes of Medicine in Jefferson Medical College, Philadelphia. With nearly 500 illustrations. Seventh edition, thoroughly revised, and extensively modified and enlarged. In 2 vols....523
General Therapeutics and Materia Medica; adapted for a medical text-book. By Robley Dunglison, M.D., &c. 182 illustrations. 4th edition, revised and improved. In 2 volumes ..................525
Elementary Chemistry, theoretical and practical. By Geo Fownes, F.R.S., Professor of Practical Chemistry in University College, London. Edited, with additions, by Robert Bridges, M. D., Professor of Chemistry in Philadelphia College of Pharmacy, etc. etc. Third American, from a late London edition. With numerous wood engravings........................526
A Practical Handbook of Medical Chemistry. By John E. Bowman, Demonstrator of Chemistry in King's College, London; and author of "Practical Chemistry .........................528
Surgical Anatomy. By Joseph Maclise, Surgeon. With colored plates. Part third ...........530
Spectacles: their Uses and Abuses in long and short sightedness; and the pathological conditions resulting from their irrational employment. By J. Sichel, M. D., of the Faculties of Berlin and Paris; Clinical Professor of Diseases of the Eye; Officer of the Legion of Honor, etc. etc. Translated from the French, by permission of the author, by Henry W. Williams, M.D., Fellow of the Massachusetts Medical Society ..................531
The American Journal of Science and Arts. Conducted by Professors B. Silliman, and B. Silliman, jr., and James D. Dana. Second series. No. 30. Nov. 1850. With an Index to Vols. I—X .........................535

S

Seguin's investigations of Peruvian bark......................471
Senega, Polygala as an emmenagogue.........................445
Single dose of quinine in treatment of ague .......................108
Singular case of monstrosity.........1
pregnancy..........7
connection of twins ...........19
Speculum practice ..............258
Spontaneous reproduction of cowpock..........................84
Spontaneous expulsion of a child..145
Statistics of mortality from fractures of the head ..............128
Surgical instruments..............460

Sutton, Dr. on typhoid fever ......273
Sutton's, Dr. report on typhoid fever ..................185, 277, 369

T

Test, hydrostatic ..................113
The American Med. Association ...85
New York Medical School ...180
Ohio Medical School .........181
Western Lancet...............181
President of the Philadelphia county Medical Society .....271
New Hampshire Med. Jour...272
New York Register of Medicine and Pharmacy .........363
use of tobacco ...............542
Western Medico-Chirurgical Journal ...................363
Western Lancet and Memphis Medical School .............366
largest liberty ...................367
Therapeutical effects of turpentine 132
Tobacco, use of...................542
Tracheotomy ......................442
Transactions of the Medical Association of Southern Central N. York..........................273
Transylvania Medical Journal .....453
Treatment of aneurism by compression.........................448
Treatment of internal hemorrhoides257
dysmenorrhea........148
Troost, Dr. death of ..............269
Tubercular exudation into the lungs ............................73
Turpentine, therapeutical effects of ..............................132
Twins singularly connected.........19
Typhoid fever report on.185, 277, 369
Typhoid fever, Becquerel's memoir on mercurial treatment of ..............................509

U

Ulceration of the lungs checked by cod-liver oil.......................73
Ulceration of the os and cervix uteri .............................258
University of Louisville ...........369
Urine, incontinence of in children.266
Use and abuse of the speculum.....457
Use of opium in obstetrics.........114
Uterine pregnancy of over twenty years endurance ................449
Uterus, excision of by Professor Paul F. Eve ......................401
Uterus, condition of soon after menstruation......................139

V

Vermifuges, remarks on............141

W

Watering streets ..................327
Webster and his spiritual adviser..150
Why epidemics rage at night ......265

Y

Yandell's, D. W. translation of M. Becquerel's memoir on the treatment of typhoid fever by mercurials.........................509

### *Receipts of Medical Journal for November*, 1850.

| | |
|---|---|
| Dr. T. N. Warfield, Hardinsburg, Ky., - - - - | $10 00 |
| H. Taylor, Owl Prairie, Ind, - - - - - | 5 00 |
| J. D. McGill, Rocky Point, Miss., - - - - | 10 00 |
| J. Z. Wheat, Columbus, Ky., - - - - - | 5 00 |
| D. Thompson, McMillins, Texas, - - - - | 5 00 |

---

### TRAVELING AGENTS FOR THE WESTERN JOURNAL OF MEDICINE AND SURGERY.

W. RAMSAY, Kentucky—C. W. JAMES Ohio, Indiana, Illinois, Missouri, and Michigan—WM. H. WELD, Louisiana and Arkansas—M. W, ROBINSON, Tennessee—H. M. LEWIS, Ala.

### DELINQUENT SUBSCRIBERS.

It will be seen that our receipts for this month are meagre indeed. Those of our subscribers who think that our Journal is not worth the money we charge for it should pay up their arrears and discontinue their subscription; if they continue their subscription they surely cannot expect us to credit them forever. Many of them are largely behind hand with us—much more we expect, in many cases, than they suppose—and we have thought we might be doing them a favor by giving them notice of the amount they are severally owing us, by publishing the same upon the cover of the Journal; if settlements are not made by our February issue we will, for their benefit, and, as we shall consider, at the request of those who have not previously made inquiry of us by letter or personally, pursue this course. We hope, however, that it may not be necessary and that all of our subscribers, who are in arrears, will take the occasion of the close of the present volume of our Journal and the approaching close of the year to close up their unpaid accounts and begin afresh.

Dec. 1850. PRENTICE & WEISSINGER.

